# Retinal microvasculature alterations are associated with mild behavioral impairment in Southeast Asian dementia‐free older adults

**DOI:** 10.1002/alz.71933

**Published:** 2026-10-30

**Authors:** Yingqi Liao, Ming Ann Sim, Jiangbo Cui, Haoran Zhang, Saima Hilal, Eddie JY Chong, Anqi Toh, Boon Yeow Tan, Narayanaswamy Venketasubramanian, Arthur Mark Richards, Xin Xu, Yih Chung Tham, Christopher Chen

**Affiliations:** ^1^ Memory, Ageing and Cognition Centre Department of Pharmacology National University of Singapore Singapore Singapore; ^2^ Department of Psychiatry School of Public Health, the Second Affiliated Hospital of School of Medicine Zhejiang University Hangzhou Zhejiang China; ^3^ Department of Intensive Care Medicine, Department of Anesthesia Ng Teng Fong General Hospital National University of Singapore, Yong Loo Lin School of Medicine Singapore Singapore; ^4^ Saw Swee Hock School of Public Health National University of Singapore Singapore Singapore; ^5^ St Luke's Hospital Singapore Singapore; ^6^ Raffles Neuroscience Centre Raffles Hospital Singapore Singapore; ^7^ Yong Loo Lin School of Medicine National University of Singapore Singapore Singapore; ^8^ National University Heart Centre Singapore Singapore; ^9^ Cardiovascular Research Institute National University of Singapore Singapore Singapore; ^10^ Christchurch Heart Institute University of Otago Christchurch Christchurch Hospital Christchurch New Zealand; ^11^ Nanhu Brain‐computer Interface Institute Hangzhou Zhejiang China; ^12^ National Key Laboratory of Brain‐Machine Intelligence Zhejiang University Hangzhou Zhejiang China; ^13^ Zhejiang Key Laboratory of Intelligent Preventive Medicine Hangzhou Zhejiang China; ^14^ Centre for Innovation and Precision Eye Health and Department of Ophthalmology Yong Loo Lin School of Medicine National University of Singapore Singapore Singapore; ^15^ Ocular Epidemiology Research Group Singapore Eye Research Institute, Singapore National Eye Centre Singapore Singapore; ^16^ Ophthalmology and Visual Science Academic Clinical Program Duke‐NUS Medical School Singapore Singapore

**Keywords:** mild behavioral impairment, neuropsychiatric symptoms, retinal imaging, retinal microvasculature

## Abstract

**BACKGROUND:**

Mild behavioral impairment (MBI) is an important neurobehavioral marker of dementia. This study examined associations between retinal vascular measures, MBI, and its potential neurobiological correlates.

**METHODS:**

Dementia‐free older adults from a memory clinic cohort were followed for up to 5 years. MBI was evaluated using the Neuropsychiatric Inventory. Retinal vessel caliber measures were quantified from fundus photographs. Associations of retinal vascular measures with neuroimaging markers of cerebral small vessel disease and plasma proteomic profiles were further explored.

**RESULTS:**

Among 302 participants, reduced arteriole‐to‐venule ratio (AVR) and narrower arteriole caliber (central retinal artery equivalent) were associated with a greater likelihood of incident MBI. Lower AVR was associated with white matter hyperintensity volume and circulating proteins related to immune function and inflammatory processes.

**CONCLUSION:**

Retinal microvascular alterations are associated with MBI in the pre‐dementia stage, with exploratory findings suggesting related vascular and inflammatory biological correlates that warrant further investigation.

## Highlights

- Retinal vascular caliber alterations are linked to behavioral impairment before dementia.
- Lower AVR and CRAE predict incident MBI.
- Lower AVR associates with WMHs and inflammation‐related proteins.

## 1 BACKGROUND

Neuropsychiatric symptoms (NPS), such as agitation, apathy, affective, and psychotic symptoms, are highly prevalent in mild cognitive impairment (∼43%) and dementia (∼75%) and have been widely recognized as early manifestations of dementia.^1^, ^2^ Accumulating evidence suggests that early NPS represent an at‐risk phenotype and are associated with a greater risk of developing dementia such as Alzheimer's disease (AD) and vascular dementia.^3^, ^4^ Therefore, understanding the underlying pathways contributing to early NPS in preclinical or prodromal stages of dementia is critical for improving early identification and informing timely preventive interventions in at‐risk older adults.

Mild behavioral impairment (MBI) has been conceptualized as an early neuropsychiatric syndrome characterized by persistent and late‐life emergent behavioral symptoms and personality change prior to dementia onset.^1^ Unlike transient NPS, which may reflect situational or reactive distress, persistent NPS meeting the MBI criteria are associated with a greater risk of cognitive decline and conversion to dementia.^5^, ^6^ Growing evidence demonstrates that MBI is associated with multiple neuropathological pathways, including amyloid and tau burden, neuroinflammation, metabolic dysfunction, and vascular dysregulation, supporting its role as a prodromal marker of neurodegenerative diseases.^7^ However, existing research has predominantly focused on AD‐related pathology, and further investigation is needed to understand how non‐AD mechanisms,^8^, ^9^ particularly vascular dysfunction, contribute to the emergence of MBI. Notably, emerging evidence suggests that vascular risk factor burden, such as diabetes and cardiovascular risk profiles, is associated with MBI and domains, especially in Asian cohorts.^5^, ^8^, ^9^, ^10^, ^11^ Neuroimaging biomarkers of cerebrovascular disease, such as white matter hyperintensities (WMHs), have also been associated with MBI.^12^, ^13^, ^14^, ^15^ These findings support a potential vascular contribution to late‐life emergent behavioral symptoms and highlight the need to further investigate vascular markers in relation to MBI.

Retinal imaging measures have been increasingly recognized as a promising non‐invasive tool for detecting pathological changes related to dementia.^16^ Embryologically, the retina is also known to share key structural and physiological features with the brain.^16^, ^17^ Retinal vascular caliber abnormalities, particularly reduced arteriolar caliber and venular widening (i.e., reduced central retinal artery equivalent [CRAE] and increased central retinal vein equivalent [CRVE]), have been associated with increased cerebral small vessel disease (CSVD) such as WMHs, systemic diseases, and cardiovascular risk factors such as diabetes and hypertension.^18^, ^19^, ^20^, ^21^, ^22^ Notably, arteriole‐to‐venule ratio (AVR) may serve as a composite marker of retinal microvascular health that is independent of the inter‐individual variation in ocular magnification, anatomy, and refractive error.^17^, ^23^, ^24^ Reduced AVR (i.e., narrower arterioles and wider venules) was associated with increased severity of psychiatric disorders such as depressive disorders and schizophrenia.^25^ However, while the microcirculation in the retina is sensitive to systemic changes including neuropsychiatric diseases,^26^ the associations of these retinal vascular measures with the progression of neuropsychiatric symptomatology in dementia‐free adults remains unknown. Addressing this gap is important for understanding neurobehavioral changes preceding dementia using accessible biomarkers. Additionally, previous neuroimaging studies highlighted the link between brain and retinal microvascular changes in AD,^27^ supporting a shared vascular basis. However, structural imaging alone may not fully capture the broader biological pathways underlying these alterations. Integrating proteomic and neuroimaging approaches would provide further insights into the vascular mechanisms contributing to early neuropsychiatric changes preceding dementia.

To this end, we examined the association between retinal vascular measures and MBI in a longitudinal Southeast Asian cohort of dementia‐free older adults. We hypothesized that narrower arteriolar, widening venular caliber, and lower AVR, reflecting greater retinal microvascular alterations, would be associated with MBI. Taken together, the findings of this study extend current evidence by highlighting the role of retinal microvascular alterations in vascular processes relevant to neuropsychiatric symptomatology in pre‐dementia individuals.

## 2 METHODS

### 2.1 Participants

This study is part of the ongoing prospective memory‐clinic cohort based in Singapore, where participants were recruited from memory clinics (National University Hospital and St Luke's Hospital) from August 2010 to 2020.^28^ Participants aged ≥50 years old who possessed sufficient language skills for neuropsychological assessments and fulfilled diagnosis criteria were eligible for study enrollment. Following the Alzheimer's Association International Society to Advance Alzheimer's Research and Treatment (ISTAART‐AA) criteria for MBI,^1^ participants were excluded in the current study if they had: (1) diagnoses of major psychiatric disorders or substance abuse disorders; (2) cognitive impairment due to traumatic brain injury, multiple sclerosis, tumor, epilepsy, or systemic diseases; (3) significant visual or auditory abnormalities affecting assessments; and (4) a diagnosis of dementia. These criteria were applied to minimize the potential misclassification of behavioral symptoms arising from primary psychiatric or other neurological or systemic conditions as MBI and to focus on late‐life behavioral symptoms occurring in the context of aging and possible neurodegenerative processes.

**RESEARCH IN CONTEXT**

1. **Systematic review**: We conducted a literature review using PubMed to examine the neurobiological underpinnings of MBI. Emerging evidence implicates vascular pathology in early behavioral disturbances preceding dementia. However, accessible biomarkers capable of capturing this substrate in clinical settings remain limited.
2. **Interpretation**: We showed that reduced CRAE and AVR were associated with incident MBI among dementia‐free older adults, highlighting retinal microvasculature alterations as potential markers of susceptibility to late‐life emergent behavioral disturbances. Additional analyses showed that lower AVR was related to neuroimaging markers of cerebrovascular disease and circulating proteins implicated in immune and inflammatory processes, providing broader biological context for vascular and inflammatory pathways potentially relevant to early neuropsychiatric manifestations.
3. **Future directions**: Future studies should corroborate our findings and integrate additional retinal imaging biomarkers as scalable, non‐invasive approaches to capture domain‐specific behavioral and cognitive changes in larger and more diverse populations.

All study participants underwent comprehensive neuropsychological tests, laboratory examination, retinal, and neuroimaging assessments at baseline and during a maximum period of 5 years of follow‐up. Baseline and longitudinal analyses were conducted using a complete‐case approach. For cross‐sectional analyses, participants were included if they had sufficient consecutive Neuropsychiatric Inventory (NPI) assessments to determine baseline MBI status and available fundus‐derived retinal vascular measures. To examine potential bias arising from missing NPI data, baseline characteristics were compared between included and excluded participants (Table S1). For longitudinal analyses, participants without baseline MBI were further required to have at least 2 years of follow‐up NPI assessments to ascertain incident MBI (Figure 1).

**FIGURE 1:**
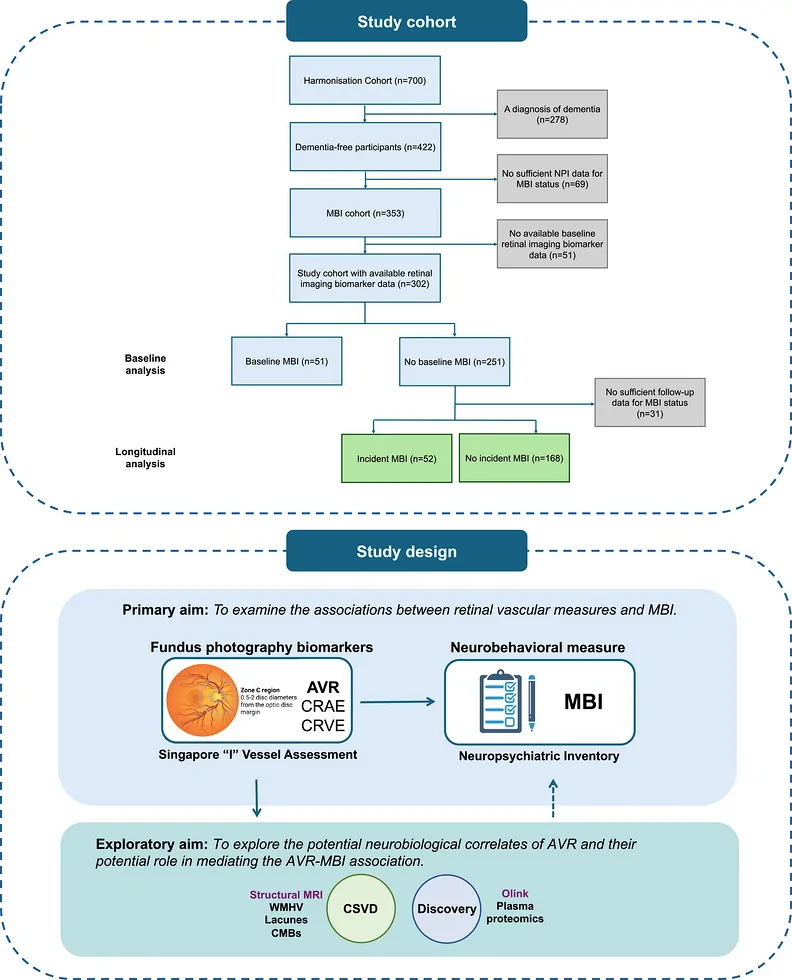
AVR, arteriole‐to‐venule ratio; CMB, cerebral microbleeds; CRAE, central retinal artery equivalent; CRVE, central retinal vein equivalent; CSVD, cerebral small vessel disease; MBI, mild behavioral impairment; NPI, Neuropsychiatric Inventory; WMHV, white matter hyperintensity volume. The retinal schematic was created at biorender.com.

Ethics approval was obtained from the National Healthcare Group Domain Specific Review Board (NHG DSRB 2018/01098 and 2010/00017). Written informed consent was obtained before study enrollment.^28^

### 2.2 Neuropsychiatric assessments

MBI was measured using the standardized NPI according to the ISTAART‐AA criteria.^5^, ^29^ Total scores of NPI were calculated by summing the severity × frequency of individual items. As the NPI has a 1‐month reference period of NPS, NPI data from consecutive annual visits were utilized to capture the persistent nature of this neurobehavioral syndrome. Specifically, baseline MBI was classified as the presence of NPS (NPI ≥ 1) across two consecutive visits at baseline and Year 1 follow‐up. For participants without baseline NPI data (*n* = 9), Years 1 and 2 were used for MBI identification.^5^, ^30^ Among those without baseline MBI, incident MBI was further identified as the emergence of NPS over any two consecutive follow‐up annual visits before conversion to dementia.^31^ The first visit at which NPS were observed was recorded as the incident event visit, while the subsequent consecutive annual visit was used to confirm symptom persistence.

### 2.3 Neuropsychological and clinical assessments

Annual assessments of cognitive tests, including Montreal Cognitive Assessment, Clinical Dementia Rating score, and a locally validated neuropsychological battery, were administered.^32^, ^33^, ^34^ Clinical diagnosis was determined based on these assessments by neurologists, psychologists, and trained research personnel at weekly consensus meetings. No cognitive impairment (NCI) was defined as no objective cognitive impairment based on the neuropsychological assessments. Cognitive impairment‐no dementia (CIND) was defined as at least one or more cognitive domain impairment without the loss of independent daily functioning. A diagnosis of dementia was given based on the *Diagnostic and Statistical Manual of Mental Disorders*, *Fourth Edition*. Details of the study procedure were previously described.^28^, ^35^ A detailed questionnaire was administered to all participants for their demographic information, including age, sex, education, and smoking status, and medical history, including cardiovascular disease, hypertension, hyperlipidemia, stroke, and diabetes mellitus, was obtained through interview and verified by a review of available medical records if available. Cardiovascular disease was defined as a medical history of myocardial infarction, congestive heart failure, atrial fibrillation, coronary angioplasty, or stenting. Apolipoprotein E (APOE) ε4 carrier status was defined by the presence of at least one APOE ε4 allele on genotyping.^36^

### 2.4 Retinal imaging measures

The full protocol for retinal fundoscopy image acquisition was described previously.^37^, ^38^ In brief, retinal photography was performed for all participants at baseline, following standardized pupillary dilation, using a 45° digital retinal camera (Canon CR‐1‐40D or Canon CR‐DGi 10D). Retinal arteriolar and venular calibers were summarized as CRAE and CRVE, as well as the AVR (i.e., CRAE/CRVE), which were obtained using the Singapore I Vessel Assessment (SIVA).^37^ In this study, caliber measures in the region within 0.5 to 2.0 optic disc diameters from the optic disc margin (Zone C) were studied as this region covers the broader retinal microvascular bed and provides a more representative estimate of arteriolar and venular calibers.^39^ SIVA automatically identified the optic disc with reference to the optic disc center and traced retinal arterioles and venules. To ensure the accuracy of automated vessel tracing, trained graders masked to participant characteristics visually reviewed the SIVA outputs and performed manual corrections where necessary, following a standardized protocol.^40^ The intra‐ and intergrader reliability of these measurements was reported previously.^40^ For primary analyses, retinal vascular measures were treated as continuous variables and standardized using the *z*‐score standardization (distribution plots of raw values shown in Figure S1).

### 2.5 Neuroimaging measures

Structural magnetic resonance imaging (MRI) scans were performed at baseline using a 3T Siemens MAGNETOM Trio TIM scanner, with a 32‐channel head coil at the National University of Singapore. The neuroimaging protocol included the following sequences: T1‐weighted, T2‐weighted, fluid‐attenuated inversion recovery (FLAIR), and susceptibility weighted images. WMH volume (WMHV) was processed by the lesion growth algorithm in the Lesion Segmentation Tool toolbox (version 3.0.0) on SPM12 using the T1‐weighted and FLAIR sequences. The total WMHV was log_10_‐transformed (log_10_ [raw WMHV +1]) for subsequent analyses due to a skewed distribution. Structural markers of CSVD, including lacunes and cerebral microbleeds (CMBs), were assessed and graded by expert researchers based on the Standards for Reporting Vascular Changes on Neuroimaging criteria,^41^ as described in detail previously.^42^

### 2.6 Proteomic analysis

Paired plasma proteomic profiling was available for 260 participants. The full experimental protocol has been described elsewhere.^43^ In brief, plasma proteomic profiling was performed using the Olink Explore 1536 platform representing diverse biological processes, yielding a final list of 1441 proteins after stringent quality control processes following our previously published approach. Protein levels were expressed as Normalized Protein eXpression values for subsequent analysis.^43^

### 2.7 Statistical analyses

Descriptive analyses comparing group differences in categorical variables and continuous variables were performed using chi‐squared tests and analysis of variance tests (or Mann‐Whitney U tests for non‐parametric data), respectively. For cross‐sectional analyses, logistic regressions were conducted to examine the associations between retinal vascular measures including CRAE, CRVE, and AVR (predictor) and baseline MBI (outcome), respectively (further sensitivity analyses detailed in Supplementary Methods). In exploratory analyses, individuals without baseline MBI were further categorized into two groups based on the same assessment window: non‐MBI NPS (i.e., NPI ≥ 1 only at one of the study visits) or the No NPS (i.e., NPI = 0 for two consecutive years) groups at baseline (Supplementary Methods).

Cox proportional hazards regression models were used to examine the associations between retinal vascular measures (CRAE, CRVE, and AVR) and incident MBI among participants free of MBI at baseline (Supplementary Methods). Participants were followed until incident MBI (first year of NPI ≥ 1 will be recorded), last available follow‐up assessment, or dementia diagnosis, whichever occurred first, with incident dementia treated as a censoring event to ensure that MBI was evaluated within the pre‐dementia stage. Models were (1) adjusted for age, sex, education, baseline cognitive status (i.e., NCI vs CIND) and (2) additionally adjusted for risk factors that were statistically significant in the group comparison (i.e., cardiovascular disease [Table 1]). Sensitivity analyses additionally adjusted for additional risk factors to assess the robustness of the findings. As additional sensitivity analyses, competing‐risk regression based on the Fine‐Gray model was conducted treating incident dementia as a competing event.

**TABLE 1:** Participant characteristics.

		Baseline outcomes			Longitudinal outcomes		
Variable	Overall (*n* = 302)	Baseline MBI (*n* = 51)	No baseline MBI (*n* = 251)	*P* ^a^	Incident MBI (*n* = 52)	No incident MBI (*n* = 168)^b^	*P* ^a^
Demographics							
Age (years, mean, SD)	71.23 (7.81), 50 to 88	72.41 (7.74), 51 to 87	70.98 (7.82), 50 to 88	0.235	74.21 (5.88), 62 to 87	70.05 (8.02), 50 to 88	**0.001**
Sex (F, *n*, %)	160 (53.0)	26 (51.0)	134 (53.4)	0.873	30 (57.7)	88 (52.4)	0.609
Education years (mean, SD)	8.40 (5.07), 0 to 23	8.75 (5.56), 0 to 22	8.33 (4.97), 0 to 23	0.592	8.37 (4.27), 0 to 18	8.62 (5.09), 0 to 23	0.745
Ethnicity (*n*, %)				0.832			0.845
Chinese	263 (87.1)	43 (84.3)	220 (87.6)		46 (88.5)	149 (88.7)	
Indian	17 (5.6)	3 (5.9)	14 (5.6)		2 (3.8)	7 (4.2)	
Malay	19 (6.3)	4 (7.8)	15 (6.0)		4 (7.7)	10 (6.0)	
Others	3 (1.0)	1 (2.0)	2 (0.8)		0 (0.0)	2 (1.2)	
Clinical, behavioral, and cognitive measures							
Diagnosis (CIND, *n*, %)	204 (67.5)	43 (84.3)	161 (64.1)	**0.008**	42 (80.8)	98 (58.3)	**0.006**
MoCA (mean, SD)	21.87 (4.76), 8 to 30	20.06 (4.99), 9 to 30	22.24 (4.64), 8 to 30	**0.003**	21.27 (4.44), 8 to 29	22.71 (4.50), 9 to 30	**0.045**
CDR‐SOB (median, IQR)	0.50(1.00), 0 to 4	1.00(1.00), 0 to 4	0(1.00), 0 to 4	**<0.001**	0.50(1.00), 0 to 3.5	0(0.50), 0 to 4	0.100
NPS status (*n*, %)				–			
No NPS	165 (54.6)	0 (0.0)	165 (65.7)		–		
Non‐MBI NPS	86 (28.5)	0 (0.0)	86 (34.3)		–		
MBI	51 (16.9)	51 (100.0)	0 (0.0)		–		
Conversion to dementia (*n*, %)	60 (19.9)	28 (54.9)	32 (12.7)	**<0.001**	–		
Risk factors							
Cardiovascular disease (*n*, %)	42 (13.9)	7 (13.7)	35 (13.9)	1.000	11 (21.2)	15 (8.9)	**0.032**
Hypertension (*n*, %)	189 (63.0)	34 (69.4)	155 (61.8)	0.395	35 (67.3)	95 (56.5)	0.223
Hyperlipidemia (*n*, %)	217 (72.1)	36 (70.6)	181 (72.4)	0.927	39 (75.0)	118 (70.7)	0.667
Stroke (*n*, %)	60 (19.9)	11 (21.6)	49 (19.6)	0.898	11 (21.6)	29 (17.3)	0.624
Diabetes mellitus (*n*, %)	88 (29.1)	22 (43.1)	66 (26.3)	**0.025**	15 (28.8)	39 (23.2)	0.522
APOE ε4 (*n*, %)	91 (30.1)	21 (41.2)	70 (27.9)	0.086	16 (30.8)	45 (26.8)	0.701
Smoking (*n*, %)	22 (7.3)	5 (9.8)	17 (6.8)	0.643	2 (3.8)	11 (6.5)	0.700
Retinal measures							
CRAE (mean, SD)^c^	111.06 (12.73), 72 to 175	107.50 (10.64), 81 to 129	111.78 (13.01), 72 to 175	**0.028**	109.38 (11.54), 81 to 151	112.91 (13.83), 72 to 175	0.096
CRVE (mean, SD)^c^	160.85 (18.11), 95 to 239	161.66 (18.17), 95 to 208	160.69 (18.13), 100 to 239	0.727	159.93 (17.03), 127 to 220	161.54 (19.47), 100 to 239	0.593
AVR (mean, SD)^c^	0.69 (0.07), 0.39 to 1.05	0.67 (0.06), 0.56 to 0.85	0.70 (0.07), 0.39 to 1.05	**0.004**	0.69 (0.06), 0.56 to 0.86	0.70 (0.07) 0.39 to 1.05	0.137
AVR status (lower, *n*, %)^d^	221 (73.2)	44 (86.3)	177 (70.5)	**0.032**	42 (80.8)	113 (67.3)	0.091
CSVD imaging biomarkers							
WMHV (mL, mean, SD)^e^, ^f^	6.72 (9.21), 0 to 60	8.63 (10.37), 0 to 45	6.33 (8.93), 0 to 60	0.062	7.39 (7.47), 0 to 31	5.33 (7.16), 0 to 40	**0.008**
Lacune count (mean, SD)^g^	0.42 (1.01), 0 to 9	0.51 (1.25), 0 to 7	0.40 (0.96), 0 to 9	0.718	0.40 (0.75), 0 to 3	0.40 (1.05), 0 to 9	0.422
CMB count (mean, SD)^g^, ^h^	1.96 (6.40), 0 to 60	2.48 (8.31), 0 to 58	1.86 (5.96), 0 to 60	0.079	2.44 (7.52), 0 to 50	1.52 (5.43), 0 to 60	0.204

Abbreviations: APOE ε4, apolipoprotein E (APOE) ε4; AVR, arteriole‐to‐venule ratio; CDR, Clinical Dementia Rating; CMB, cerebral microbleed; CRAE, central retinal artery equivalent; CRVE, central retinal vein equivalent; CSVD, cerebral small vessel disease; F, female; IQR, interquartile range; MoCA, Montreal Cognitive Assessment; SD, standard deviation; WMHV, white matter hyperintensity volume.

^a^ Group differences in categorical variables and continuous variables were examined using chi‐squared tests and ANOVA tests for parametric data or Mann‐Whitney U tests for non‐parametric data. Ranges for continuous variables are additionally reported.

^b^ Among the 168 individuals without incident MBI, 18 developed incident dementia during follow‐up without a preceding MBI episode and were censored at dementia diagnosis, while 150 remained free of both incident MBI and dementia. Given that dementia precludes the ascertainment of incident MBI, these individuals were further accounted for as competing events in sensitivity analyses using competing risk models.

^c^ Raw central retinal arteriolar and venular equivalents (CRAE, CRVE) and AVR values were reported for descriptive analyses, and z‐score‐transformed values were used as continuous variables for statistical analyses.

^d^ Lower AVR is defined based on ≤75th percentile; ≤0.73.

^e^ Biomarker measures showed a skewed distribution. Mean and SD of biomarker measures were reported, while group comparisons were performed in log_10_‐transformed biomarker levels.

^f^ 297 individuals with available WMHV data.

^g^ CSVD biomarker contains a large proportion of zeros, resulting in a median of zero or one. Thus, mean and standard deviation were presented to better illustrate the biomarker level. Group comparisons were performed using non‐parametric Mann‐Whitney U tests.

^h^ 299 individuals with available CMB data.

Models assessing CRAE or CRVE in relation to MBI were additionally adjusted for the fellow vessel calibers to account for the shared variation between arteriolar and venular measures.^44^, ^45^ For ease of interpretability, effect estimates were reported per standard deviation (SD) decrease in AVR and CRAE and per SD increase in CRVE, such that higher estimates represent poorer microvascular health (narrower arteriolar caliber, widening venular caliber, and lower AVR) and a greater likelihood of MBI. Additionally, to assess robustness for AVR analyses, analyses were repeated using AVR quartiles. Based on the observed distribution and quartile patterns, AVR was further dichotomized at the 75th percentile (lower AVR ≤75th [0.73] vs higher AVR > 75th percentile) to facilitate clinical interpretation. The dichotomized AVR variable was additionally used for visualization and for subsequent analyses examining associations with neuroimaging and proteomic markers.

We further performed analyses to examine the associations between neuropathological biomarkers and lower AVR (≤75th percentile; ≤0.73). Linear regression was individually fitted to understand the associations between lower AVR and log_10_ WMHV, while negative binomial regression was fitted to evaluate the associations between lower AVR with lacune and CMB count, respectively. These models were adjusted for age and sex.

To further understand the biological underpinnings of AVR, we performed discovery analyses using paired plasma proteomic data. Logistic regression models were performed to study the protein levels against lower AVR, adjusted for age and sex. *P* values were reported with correction for multiple testing using the *q* value method, with statistical significance calls made at *p* < 0.05 and *q* < 0.1.^46^ We visualized the top 10 proteins associated with lower AVR using volcano plots. Over‐representation analyses of all significant proteins associated with lower AVR were performed using the DAVID bioinformatics resource, mapped to Reactome, Gene Ontology Biological Processes, and Kyoto Encyclopedia of Genes and Genomes pathways. *P* values were subject to false discovery rate correction using the Benjamini‐Hochberg method. Finally, we performed exploratory mediation analyses to evaluate whether the neuroimaging biomarkers or plasma proteins significantly associated with lower AVR mediated the observed association with incident MBI, using the regmedint package in R. Models were adjusted for age and sex.

All statistical analyses were conducted using R version 4.4.2 (2024‐10‐31) (RStudio 2024.12.0). Significant results of statistical tests were set as *p <* 0.05 unless otherwise stated.

## 3 RESULTS

### 3.1 Participant characteristics

A total of 302 dementia‐free participants with available MBI information and retinal imaging biomarkers were included in the current study. At baseline, 51 (16.9%) participants had MBI. Among 220 participants without baseline MBI and available follow‐up data, we identified 52 (23.6%) participants who developed incident MBI over a median of 4 (interquartile range: 2 to 4) years (Figure 1). Participant characteristics are summarized in Table 1.

### 3.2 Cross‐sectional and longitudinal associations between retinal vascular measures and MBI

In cross‐sectional analyses, lower AVR, lower CRAE, and higher CRVE were independently associated with baseline MBI, both when compared with participants without MBI (Table S2) and when compared with participants without NPS (Table S3). When further extending this to longitudinal observation, we showed that a lower AVR (per SD decrease) was significantly predictive of a greater likelihood of incident MBI (hazard ratio [HR] = 1.34, 95% confidence interval [CI] = 1.03 to 1.75, *p =* 0.030) (Table 2; Figure 2). Notably, only lower CRAE remained independently associated with incident MBI (HR = 1.46, 95% CI = 1.04 to 2.05, *p =* 0.027), while CRVE was not significant (HR = 1.29, 95% CI = 0.93 to 1.80, *p =* 0.127) (Table 2), suggesting that arteriolar narrowing may specifically contribute to the longitudinal AVR–MBI relationship. In the sensitivity analyses, this longitudinal relationship remained significant for AVR (subdistribution HR [SHR] = 1.35, 95% CI = 1.07 to 1.71, *p =* 0.011) and CRAE (SHR = 1.47, 95% CI = 1.10 to 1.98, *p =* 0.009) when accounting for incident dementia as the competing risk event (Table S4).

**TABLE 2:** Cox regression on the associations between retinal vascular measures and incident mild behavioral impairment.

		Incident MBI	
	**Model 1**	**Model 2**	**Model 3**
Retinal vascular measure	**HR (95% CI)**	**HR (95% CI)**	**HR (95% CI)**
AVR (per 1‐SD decrease)	1.22 (0.95 to 1.56)	**1.36 (1.04 to 1.78)**	**1.34 (1.03 to 1.75)**
CRAE^a^ (per 1‐SD decrease)	**1.36 (1.01 to 1.85)**	**1.50 (1.07 to 2.09)**	**1.46 (1.04 to 2.05)**
CRVE^b^ (per 1‐SD increase)	1.07 (0.78 to 1.48)	1.31 (0.94 to 1.82)	1.29 (0.93 to 1.80)

*Note*: Retinal biomarkers were z‐transformed. *P < 0.05 is highlighted in bold.

Abbreviations: AVR, arteriole‐to‐venule ratio; CI, confidence interval; CRAE, central retinal artery equivalent; CRVE, central retinal vein equivalent; SD, standard deviation.

^a^ Adjusted for CRVE in model.

^b^ Adjusted for CRAE in model.

Model 1: null.

Model 2: age + sex + education years + baseline cognitive status (no cognitive impairment vs cognitive impairment‐no dementia).

Model 3: Model 2 + cardiovascular disease.

**FIGURE 2:**
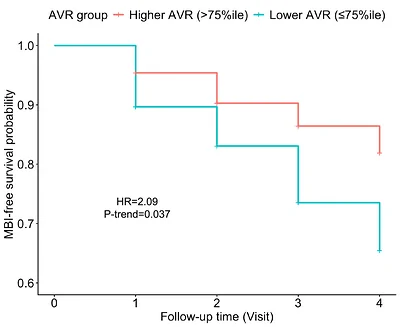
Association between AVR and incident MBI. The Kaplan‐Meier Curve for incident MBI stratified by lower (≤75th percentile; ≤0.73) and higher (>75th percentile; > 0.73) AVR. Estimates and *p* value for the unadjusted model are presented in the figure, with adjusted model results (*p <* 0.05) presented in Table S5. AVR, arteriole‐to‐venule ratio; HR, hazard ratio; MBI, mild behavioral impairment.

Similar patterns were observed when examining AVR categorically, with both quartile‐based and dichotomized AVR (≤75th vs > 75th percentile) demonstrating consistent associations with baseline and incident MBI (Figure S2; Tables S4 and S5). The associations between retinal vascular measures and MBI remained consistent after additional adjustment for APOE ε4 status and hypertension (Table S6) and after accounting for assessment timing (Table S7).

### 3.3 Exploratory analyses of neurobiological correlates of AVR and their potential role in mediating AVR–MBI association

We further examined the associations between neurobiological biomarkers with lower AVR to explore the potential biological correlates of retinal microvascular changes. Lower AVR was associated with a greater WMHV (*β* = 0.14, 95% CI = 0.04 to 0.25, *p =* 0.007) (Figure 3A; Table S8). Additionally, discovery analyses showed that lower AVR was associated with 78 protein signatures of vascular integrity, angiogenesis, immune/inflammation, neurodegeneration, and synaptic function (Figure 3B,C; Table S9). In exploratory mediation analyses, we did not observe a significant mediation effect of WMHV on the AVR–incident MBI association. Of the 78 proteins examined, five (IGF1R, DTX3, NCS1, GPR37, C2) showed suggestive mediation signals (*p <* 0.05) (Table S10).

**FIGURE 3:**
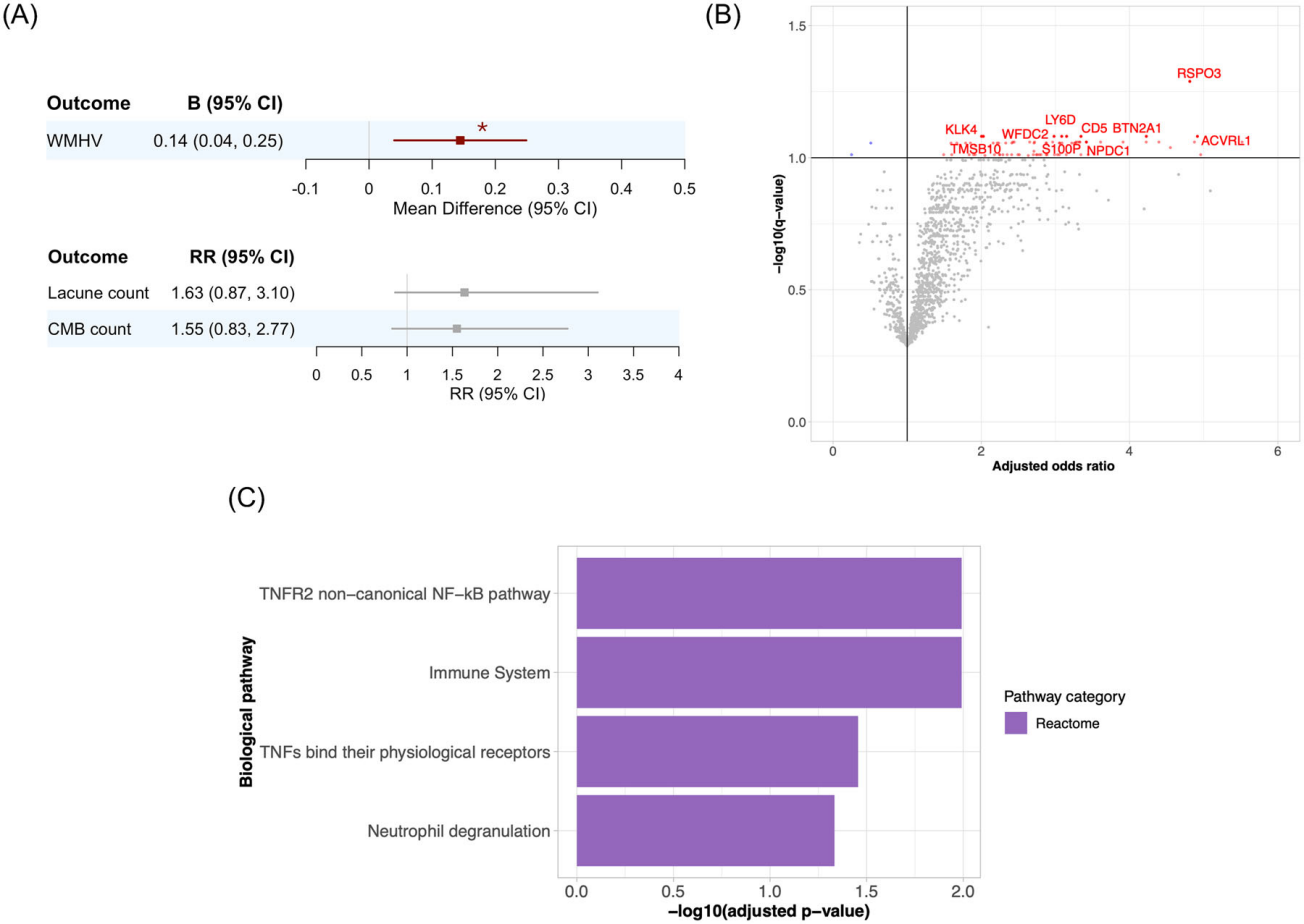
Associations between neuroimaging biomarkers and proteomic profile of lower AVR. (A) Associations between lower AVR (≤75th percentile; ≤0.73) and neuroimaging biomarkers. Significant results with *p <* 0.05 are highlighted with asterisk. (B) Top 10 (of 78 significant) plasma proteins associated with lower AVR group. *P* values were corrected for *q* value correction, with *p* < 0.05, *q* < 0.1 taken as the statistically significant threshold. (C) Significantly over‐represented biological pathways associated with lower AVR include immune and inflammatory processes.

## 4 DISCUSSION

We found that lower AVR was associated with incident MBI among dementia‐free older adults in a well‐characterized Asian memory clinic cohort, suggesting retinal microvasculature abnormalities, specifically narrower arterioles, may reflect susceptibility to emergent behavioral disturbances preceding dementia onset. Utilizing neuroimaging and plasma proteomic data, exploratory analyses suggested that lower AVR was associated with greater WMH burden and inflammation‐related proteins. These findings highlight that microvascular health may warrant further investigation as a low‐cost, non‐invasive marker of neurobehavioral risk in the pre‐dementia stage.

Dementia may manifest with early behavioral changes, sometimes even before cognitive impairment, and early NPS may share underlying neuropathology with the later dementia stages.^1^, ^47^, ^48^ Previous studies showed that retinal imaging captured microvascular abnormalities in preclinical and symptomatic AD patients, highlighting the link between retinal vascular alterations and cognitive decline.^16^, ^49^ Additionally, retinal vascular caliber has been associated with multiple adverse outcomes including incident dementia,^50^, ^51^ cardiovascular diseases,^52^ and stroke.^53^ Our longitudinal findings further showed that lower AVR, particularly narrower arteriolar caliber (CRAE), could serve as baseline predictors of incident MBI among participants who were MBI‐free at baseline. Furthermore, we found that narrower arterioles and wider venules, captured by lower AVR, were associated with prevalent MBI, independent of baseline cognitive status. Notably, this relationship was only significant between MBI (i.e., persistent NPS) and the No NPS group, but not between non‐MBI NPS (i.e., transient NPS) and No NPS. This pattern suggests that the observed association may be more closely linked to the persistent behavioral phenotype and may not be fully attributable to generalized vascular aging alone. Future studies comparing other neurocognitive and neuropsychiatric conditions are needed to establish whether these changes are specific to persistent neurobehavioral disturbances rather than broader vascular aging.

Our findings are also consistent with previous studies in which narrower arteriolar caliber was predictive of incident dementia among CIND subjects.^51^ Retinal arteriolar narrowing has been linked to hypertension,^22^, ^24^ which may impair cerebral vascular autoregulation and contribute to hypoperfusion and CSVD‐related processes.^54^ Moreover, recent resting‐state MRI evidence suggested that both narrower CRAE and lower AVR were negatively associated with the nodal local efficiency of functional connectivity in the left orbital gyrus,^55^ a region involved in affective regulation, impulse control, and self‐monitoring networks,^56^, ^57^ which are core domains affected in MBI. Notably, retinal vascular caliber measures primarily reflect structural vascular remodeling rather than directly assessing dynamic microvascular function.^58^, ^59^ Although MBI represents an early clinical manifestation along the dementia continuum, the observed association may reflect accumulated vascular burden rather than early microvascular dysfunction among dementia‐free older adults.

Retinal vascular biomarkers are associated with stroke and cardiovascular diseases,^19^, ^21^ as well as neuroimaging biomarkers of CSVD and brain atrophy.^18^, ^60^ Consistent with previous literature, our study further showed that lower AVR was associated with WMHV, supporting the association between retinal microvascular changes and cerebral vascular burden in pre‐dementia populations. However, findings remained inconsistent on the links between vascular pathology such as CSVD with the emergence and progression of NPS in the community and memory‐clinic cohorts, with some studies reporting greater WMHV and increased CSVD burden among individuals with MBI^13^, ^15^ but others not reporting the observed relationship.^61^ In our present study, WMHV showed a trend toward association with MBI but did not significantly mediate the AVR–incident MBI association. These findings suggest that the AVR–MBI association may reflect vascular processes beyond WMH burden alone. Lower AVR may represent accumulated systemic vascular remodeling associated with susceptibility to MBI, rather than being primarily reflective of CSVD pathology.

Our exploratory analysis using plasma proteomics further showed that lower AVR was linked with proteins implicated in several pathophysiological processes, including angiogenesis and vascular regulation (ACVRL1,^62^ RSPO3), immune regulation and inflammation (CD5,^63^ BTN2A1, LY6D), neuronal signaling (TMSB10), and cell proliferation (S100P,^64^ WFDC2, KLK4). Additionally, the 78 proteins significantly associated with lower AVR were further mapped on pathways implicated in immune and inflammatory processes, consistent with the known associations of inflammation and immune dysfunction in microvascular health, which have been implicated in cognitive impairment and dementia.^65^, ^66^, ^67^, ^68^ These exploratory findings provide preliminary biological context for AVR–MBI association by identifying CSVD‐ and inflammation‐related pathways. However, larger studies are required to validate these preliminary observations and further elucidate their biological relevance.

Additionally, AVR is a first‐generation retinal vascular metric that provides a global summary of arteriolar‐to‐venular caliber and may not fully capture the complexity of retinal microvascular pathology relevant to neurodegeneration. Future studies should incorporate multimodal biomarkers to examine the retina–brain relationship in capturing vascular and inflammation pathology for monitoring disease progression. Furthermore, CRAE and CRVE are quantitative measures of major retinal arteriolar and venular caliber and do not capture capillary‐level pathology, which is closely linked to neurodegenerative processes.^16^ For instance, recent optical coherence tomography angiography‐derived vessel measures on capillary changes and deep‐learning approaches yielded retinal biomarkers that predicted cognitive decline and dementia risk.^38^, ^69^ These approaches further offer a non‐invasive way to characterize vascular and neurodegenerative vulnerability through the retina–brain axis. Given that both neurocognitive and neurobehavioral impairment could concurrently contribute to worse prognosis in neurodegenerative diseases, future studies should evaluate whether such composite retinal aging could also capture the vulnerability to NPS disturbances across the dementia continuum.

The strength of this study is its highlighting that retinal vascular alterations were associated with persistent behavioral changes and, more importantly, predictive of the emergence of behavioral symptoms among dementia‐free older adults. Our findings support further investigation of retinal vascular measures as lower‐cost and non‐invasive markers for vascular susceptibility associated with neurobehavioral impairments, especially in those low‐ and middle‐income countries where neuroimaging resources are limited.^70^ However, our study is not without limitations. First, longitudinal data for retinal vascular imaging scans were not available for this study. Thus, retinal vascular measures should be considered as baseline vascular markers associated with baseline MBI and subsequent risk of incident MBI. In addition, baseline MBI classification requires NPI information across baseline and Y1 to approximate symptom persistence, whereas retinal imaging was measured at baseline. This may introduce a slight temporal mismatch in the cross‐sectional analyses, and the observed association should be interpreted cautiously and not be taken as evidence of temporal directionality. Nevertheless, the primary longitudinal analyses examined baseline retinal vascular measures as predictors of incident MBI among participants who were MBI‐free at baseline, supporting the potential prognostic relevance of baseline AVR and CRAE, although this does not establish their ordering relative to cerebral vascular pathology. Future studies incorporating repeated retinal imaging and neuroimaging assessments are needed to clarify the temporal relationships between retinal vascular changes, cerebral vascular pathology, and the emergence and progression of behavioral symptoms. Second, pupil dilation before retinal imaging measures was employed. Although dilation may improve image quality and measurement precision, prior evidence suggested that it could systematically affect retinal vessel caliber measurement, which may in turn influence derived AVR estimates.^71^ In this study, dilation was applied consistently across all participants, which might have reduced differential measurement bias between individuals. Future studies should seek to replicate the observed associations using imaging protocols that do not require pupil dilation. Third, our memory‐clinic cohort may limit the generalizability of the findings to community‐dwelling older adults. In addition, although NPI has been widely used in existing cohorts to identify MBI, it was originally designed for assessing NPS in dementia patients and was not specifically developed for pre‐dementia populations. As a proxy measure for MBI, its 1‐month reference period may also capture transient NPS rather than persistent late‐life behavioral changes. Moreover, as MBI ascertainment using the NPI requires consecutive assessments to approximate symptoms persistence, participants with insufficient longitudinal NPI data were excluded. While baseline demographic and cognitive characteristics were broadly comparable between participants with and without sufficient NPI assessments, potential selection bias related to longitudinal follow‐up completion cannot be fully excluded. Hence, future large‐scale community studies are needed to identify MBI using other tools such as the MBI Checklist,^72^ which may more directly capture persistent late‐life behavioral changes before dementia onset.

In conclusion, retinal vascular caliber alterations are associated with MBI cross‐sectionally and longitudinally among older adults without dementia. Retinal vascular caliber may represent one component of systemic vascular remodeling associated with MBI. However, further research on more sensitive retinal biomarkers is likely needed before retinal imaging can be considered a clinically useful biomarker of late‐life emergent neurobehavioral disturbances preceding dementia onset.

## CONFLICT OF INTEREST STATEMENT

The authors declare no conflicts of interest. Author disclosures are available in the Supporting Information.

## CONSENT STATEMENT

Written informed consent was obtained from study participants before study enrollment.

## Supporting information




**Supporting Information**: alz71933‐sup‐0001‐SupMat.docx

**Supporting Information**: alz71933‐sup‐0002‐ICMJE.pdf
