# Supplementary material for: Retinal microvasculature alterations are associated with mild behavioral impairment in Southeast Asian dementia‐free older adults
Source: Alzheimers Dement. 2026 Oct 30;22(10):e71933. doi: 10.1002/alz.71933 (PMC13722608; doi:10.1002/alz.71933)
Supplement: Supplementary file 2 [file ALZ-22-e71933-s001.docx]

**Supplementary Methods**

**Statistical analyses**

1. Cross-sectional analyses between retinal vascular measures and baseline MBI

For cross-sectional analyses, logistic regressions were conducted to examine the associations between AVR (predictor) and baseline MBI (outcome). Models were first adjusted for 1) age, sex, education, baseline cognitive status (i.e. NCI vs CIND); and 2) additionally adjusted for risk factors that were statistically significant in the group comparison (i.e. diabetes mellitus (Table 1)). Sensitivity analyses additionally adjusted for APOE ε4 status and hypertension to assess the robustness of the findings. Further sensitivity analyses were performed to adjust for the years of NPI data used for MBI ascertainment and the interval between retinal and neuropsychological assessments to account for potential variability in assessment timing.

For exploratory analyses, individuals without baseline MBI were further categorized into two groups based on the same assessment window: non-MBI NPS (i.e. NPI≥1 only at one of the study visits) or no NPS (i.e. NPI = 0 for two consecutive years) groups at baseline. Multinomial logistic regression models were fitted to examine the association between retinal vascular measures (expressed in z scores) and baseline MBI status (three groups), with non-MBI NPS and no NPS group treated as the reference group in separate models.

2. Longitudinal analyses between retinal vascular measures and incident MBI

The median (IQR) follow-up time was estimated using the reverse Kaplan-Meier method. All exposures and covariates entered in the cox regression models were measured at baseline and treated as time-independent variables throughout follow-up. The proportional hazards assumption was assessed using Schoenfeld residuals for each cox model, and residual plots were visually examined to verify that the hazards remained proportional over time. Additionally, given that dementia precludes the ascertainment of incident MBI, these individuals were further accounted for as competing events in sensitivity analyses using competing risk models.

3. Analyses between retinal vascular measures and neuroimaging measures

Sensitivity analyses were further performed to adjust for the interval between retinal and neuroimaging assessments to account for variability in assessment timing.

**Supplementary Tables**

Supplementary Table 1. Participant characteristics between participants with and without sufficient NPI assessment.

|  | **Sufficient NPI assessment for MBI status**  **(n=353)** | **No sufficient NPI assessment**  **(n=69)** | **P** |
| --- | --- | --- | --- |
| **Demographics** |  |  |  |
| Age (mean, SD) | 71.63 (7.83) | 71.71 (8.38) | 0.940 |
| Sex (F, n, %) | 1.53 (0.50) | 1.57 (0.50) | 0.620 |
| Education years (mean, SD) | 8.20 (5.09) | 8.07 (5.15) | 0.852 |
| Ethnicity (n, %) |  |  | 0.281 |
| Chinese | 308 (87.3) | 56 (81.2) |  |
| Indian | 21 (5.9) | 7 (10.1) |  |
| Malay | 21 (5.9) | 4 (5.8) |  |
| Others | 3 (0.8) | 2 (2.9) |  |
| **Clinical, behavioral and cognitive measures** | | | |
| Diagnosis (CIND, n, %) | 0.69 (0.46) | 0.67 (0.47) | 0.723 |
| MoCA (mean, SD) | 21.64 (4.84) | 21.41 (5.53) | 0.723 |
| CDR-SOB (median, IQR) | 0.5(1.0) | 0.5(1.0) | 0.748 |
| Conversion to dementia (n, %) | 0.22 (0.42) | 0.12 (0.33) | 0.090 |

*P<0.05 are highlighted in bold.

Supplementary Table 2. Logistic regression on the associations between retinal vascular measures and baseline MBI.

|  | **Baseline MBI** | | | | | |
| --- | --- | --- | --- | --- | --- | --- |
|  | **Model 1** |  | **Model 2** |  | **Model 3** |  |
|  | **OR (95%CI)** | **P** | **OR (95%CI)** | **P** | **OR (95%CI)** | **P** |
| AVR (per 1-SD decrease) | 1.62 (1.17-2.27) | **0.004** | 1.72 (1.23-2.44) | **0.002** | 1.71 (1.22-2.44) | **0.002** |
| CRAE^a^ (per 1-SD decrease) | 1.92 (1.28-2.98) | **0.002** | 2.02 (1.32-3.15) | **0.001** | 2.03 (1.32-3.18) | **0.001** |
| CRVE^b^ (per 1-SD increase) | 1.58 (1.06-2.40) | **0.027** | 1.73 (1.15-2.66) | **0.010** | 1.72 (1.13-2.64) | **0.013** |

Retinal biomarkers were z-transformed. *P<0.05 are highlighted in bold.

a. Adjusted for CRVE in the model.
b. Adjusted for CRAE in the model.

Model 1: null

Model 2: age + sex + education years + baseline cognitive status (NCI vs CIND)

Model 3: Model 2 + diabetes mellitus

Supplementary Table 3. Multinomial logistic regression on the associations between retinal vascular measures with baseline MBI three group (baseline MBI, non-MBI NPS and no NPS).

|  | Baseline MBI vs No NPS | | Baseline MBI vs Non-MBI NPS | | Non-MBI NPS vs No NPS | |
| --- | --- | --- | --- | --- | --- | --- |
|  | **RRR (95%CI)** | **P** | **RRR (95%CI)** | **P** | **RRR (95%CI)** | **P** |
| Model 1 |  |  |  |  |  |  |
| AVR (per 1 – SD decrease) | 1.75 (1.22-2.51) | **0.002** | 1.65 (1.12-2.42) | **0.011** | 1.06 (0.81-1.39) | 0.664 |
| Model 2^a^ |  |  |  |  |  |  |
| CRAE (per 1 – SD decrease) | 2.05 (1.30-3.23) | **0.002** | 1.97 (1.21-3.22) | **0.007** | 1.04 (0.73-1.49) | 0.827 |
| Model 3^b^ |  |  |  |  |  |  |
| CRVE (per 1 – SD increase) | 1.79 (1.14-2.79) | **0.011** | 1.60 (0.99-2.59) | 0.053 | 1.11 (0.78-1.60) | 0.557 |

Relative risk ratio (RRRs) were estimated using multinomial logistic regression with ‘No NPS’ or ‘Transient NPS’ as the reference category. An RRR > 1 indicates a higher likelihood of being in the comparison group relative to the reference group, per 1-SD decrease in CRAE and AVR or increase in CRVE. Model adjusted for age, sex, education, diagnostic group and diabetes mellitus. *P<0.05 are highlighted in bold.

a. Additionally adjusted for CRVE.
b. Additionally adjusted for CRAE.

Supplementary Table 4. Competing risk regression on the associations between retinal vascular measures with incident MBI.

|  | Incident MBI |  |
| --- | --- | --- |
|  | **SHR** (**95%CI**) | **P** |
| Model 1 |  |  |
| AVR (per 1 – SD decrease) | 1.35 (1.07-1.71) | **0.011** |
| Model 2^a^ |  |  |
| CRAE (per 1 – SD decrease) | 1.47 (1.10-1.98) | **0.009** |
| Model 3^b^ |  |  |
| CRVE (per 1 – SD decrease) | 1.31 (0.98-1.74) | 0.068 |
| Model 4^c^ |  |  |
| AVR quartiles |  | **0.018^a^** |
| AVR Q1 ^d^ | 2.53 (1.06-6.02) | **0.036** |
| AVR Q2 ^d^ | 2.54 (1.12-5.72) | **0.025** |
| AVR Q3 ^d^ | 1.93 (0.86-4.30) | 0.110 |
| Model 5 ^d^ |  |  |
| Lower AVR (≤75th percentile) | 2.36 (1.20-4.66) | **0.013** |

Given the conceptualization of MBI as a pre-dementia syndrome, incident dementia occurring prior to any behavioral symptoms or among individuals without incident MBI was treated as a competing event that precludes MBI diagnosis. Model adjusted for age, sex, education, diagnostic group and cardiovascular disease at baseline. *P<0.05 are highlighted in bold.

a. Additionally adjusted for CRVE.
b. Additionally adjusted for CRAE.
c. P- trend for AVR quartiles is reported.
d. Reference group: Higher AVR group (Q4, >75^th^ percentile; >0.73).

Supplementary Table 5. Association between lower AVR (quartile-based) with baseline and incident MBI.

|  | Baseline MBI^a^ | | Incident MBI^b^ | |
| --- | --- | --- | --- | --- |
|  | **OR (95%CI)** | **P** | **HR (95%CI)** | **P** |
| Model 1 |  |  |  |  |
| AVR quartiles^c^ |  | **0.001** |  | **0.022** |
| AVR Q1^d^ | 4.41 (1.76-12.26) | **0.002** | 2.58 (1.09-6.10) | **0.031** |
| AVR Q2 ^d^ | 2.23 (0.83-6.44) | 0.119 | 2.64 (1.15-6.07) | **0.022** |
| AVR Q3 ^d^ | 1.39 (0.48-4.18) | 0.541 | 2.17 (0.93-5.03) | 0.072 |
| Model 2 ^d^ |  |  |  |  |
| Lower AVR (≤75th percentile) | 2.87 (1.28-7.39) | **0.017** | 2.42 (1.20-4.85) | **0.013** |

*P<0.05 are highlighted in bold.

a. No baseline MBI was treated as the reference group in the logistic regression model. Model adjusted for age, sex, education, diagnostic group and diabetes.
b. No incident MBI was treated as the reference group in the cox regression model. Model adjusted for age, sex, education, diagnostic group and cardiovascular disease
c. P- trend for AVR quartiles is reported.
d. Reference group: Higher AVR group (Q4, >75^th^ percentile; >0.73)

Supplementary Table 6. Associations between retinal vascular measures with baseline and incident MBI with additional covariate adjustment.

|  | Baseline MBI ^a^ | | | | Incident MBI ^b^ | | | |
| --- | --- | --- | --- | --- | --- | --- | --- | --- |
|  | **Model 1** |  | **Model 2** |  | **Mode 1** |  | **Model 2** |  |
|  | **OR (95%CI)** | **P** | **OR (95%CI)** | **P** | **HR (95%CI)** | **P** | **HR (95%CI)** | **P** |
| AVR (per 1 – SD decrease) | 1.71 (1.22-2.44) | **0.002** | 1.64 (1.16-2.35) | **0.006** | 1.34 (1.03-1.75) | **0.030** | 1.33 (1.01-1.75) | **0.043** |
| CRAE ^c^ (per 1 – SD decrease) | 2.03 (1.32-3.18) | **0.001** | 1.92 (1.25-3.04) | **0.004** | 1.46 (1.04-2.05) | **0.027** | 1.47 (1.03-2.09) | **0.035** |
| CRVE ^d^ (per 1 – SD increase) | 1.72 (1.13-2.64) | **0.013** | 1.66 (1.08-2.57) | **0.022** | 1.29 (0.93-1.80) | 0.127 | 1.28 (0.91-1.79) | 0.151 |

*P<0.05 are highlighted in bold.

a. Cross-sectional analyses

Model 1: adjusted for age, sex, education, diagnostic group, diabetes mellitus

Model 2: Model 1 + adjusted for APOE ε4 + hypertension

b. Longitudinal analyses

Model 1: adjusted for age, sex, education, diagnostic group, cardiovascular disease

Model 2: Model 1 + adjusted for APOE ε4 + hypertension

c. Additionally adjusted for CRVE.
d. Additionally adjusted for CRAE.

Supplementary Table 7. Associations between retinal vascular measures with baseline and incident MBI after additionally adjusting for assessment timing.

|  | Baseline MBI | | | | Incident MBI | |
| --- | --- | --- | --- | --- | --- | --- |
|  | **Model 1** |  | **Model 2** |  | **Model 3** |  |
|  | **OR (95%CI)** | **P** | **OR (95%CI)** | **P** | **HR (95%CI)** | **P** |
| AVR (per 1-SD decrease) | 1.72 (1.22 – 2.46) | **0.003** | 1.73 (1.22 – 2.47) | **0.002** | 1.37 (1.04 – 1.81) | **0.024** |
| CRAE^a^ (per 1-SD decrease) | 2.02 (1.31 – 3.16) | **0.002** | 2.04 (1.33 – 3.21) | **0.001** | 1.47 (1.04 -2.09) | **0.029** |
| CRVE^b^ (per 1-SD increase) | 1.75(1.14 – 2.71) | **0.010** | 1.74 (1.14 – 2.70) | **0.011** | 1.36 (0.96 – 1.92) | 0.084 |

No baseline MBI was treated as the reference group in the logistic regression model. Retinal biomarkers (z-transformed) were entered in the model separately. *P<0.05 are highlighted in bold.

a. Adjusted for CRVE in the model.
b. Adjusted for CRAE in the model.

Model 1: adjusted for age, sex, education, diagnostic group, diabetes and years used for MBI diagnosis (Year 1/Year 2).

Model 2: adjusted for age, sex, education, diagnostic group, diabetes, years used for MBI diagnosis and intervals between assessments (in months).

Model 3: adjusted for age, sex, education, diagnostic group, cardiovascular disease and years used for MBI diagnosis (Year 1/Year 2).

Supplementary Table 8. Associations between lower AVR with neuroimaging measures after additionally adjusting for assessment timing.

|  | WMHV |  | Lacunes |  | CMB |  |
| --- | --- | --- | --- | --- | --- | --- |
|  | **β (95%CI)** | **P** | **RR (95%CI)** | **P** | **RR (95%CI)** | **P** |
| AVR (per 1-SD decrease) | 0.14 (0.04 – 0.25) | **0.008** | 1.80 (0.94 – 3.48) | 0.078 | 1.53 (0.82 – 2.72) | 0.158 |

Linear regression was used to examine the association between lower AVR (≤75th percentile) and log10-transformed white matter hyperintensity volume (WMHV), while separate negative binomial regression models were used to examine the associations of lower AVR with lacune count and cerebral microbleed (CMB) count. All models were adjusted for age, sex and assessment intervals between retinal and neuroimaging assessments. *P<0.05 are highlighted in bold.

Supplementary Table 9. All plasma proteins significantly associated with lower AVR.

| **OlinkID** | **Assay** | **Description** | **OR** | **95% CI LL** | **95% CI UL** | **p-value** | **q-value** |
| --- | --- | --- | --- | --- | --- | --- | --- |
| OID21415 | RSPO3 | R-spondin-3 | 4.812926 | 2.219828 | 10.43516 | 6.9E-05 | 0.051398 |
| OID21505 | WFDC2 | WAP four-disulfide core domain protein 2 | 2.981642 | 1.613988 | 5.508214 | 0.000485 | 0.082978 |
| OID20999 | ACVRL1 | Serine/threonine-protein kinase receptor R3 | 4.915599 | 1.972139 | 12.25224 | 0.000632 | 0.082978 |
| OID20708 | LY6D | Lymphocyte antigen 6D | 3.084949 | 1.609533 | 5.912839 | 0.000689 | 0.082978 |
| OID21391 | KLK4 | Kallikrein-4 | 2.003001 | 1.340186 | 2.993625 | 0.000704 | 0.082978 |
| OID20103 | S100P | Protein S100-P | 3.151095 | 1.613733 | 6.153063 | 0.000775 | 0.082978 |
| OID21449 | CD5 | T-cell surface glycoprotein CD5 | 3.344741 | 1.647109 | 6.79208 | 0.000836 | 0.082978 |
| OID20713 | BTN2A1 | Butyrophilin subfamily 2 member A1 | 4.226235 | 1.800521 | 9.919943 | 0.00093 | 0.082978 |
| OID21118 | TMSB10 | Thymosin beta-10 | 2.02813 | 1.330856 | 3.090726 | 0.001003 | 0.082978 |
| OID20205 | NPDC1 | Neural proliferation differentiation and control protein 1 | 3.417161 | 1.601262 | 7.292367 | 0.001486 | 0.087249 |
| OID21001 | ADAM22 | Disintegrin and metalloproteinase domain-containing protein 22 | 3.325504 | 1.577878 | 7.008763 | 0.001583 | 0.087249 |
| OID20764 | PLAUR | Urokinase plasminogen activator surface receptor | 3.60893 | 1.617923 | 8.050059 | 0.001716 | 0.087249 |
| OID21458 | NCS1 | Neuronal calcium sensor 1 | 4.225162 | 1.714149 | 10.41449 | 0.001743 | 0.087249 |
| OID20935 | EFNA4 | Ephrin-A4 | 3.278138 | 1.545179 | 6.954657 | 0.001975 | 0.087249 |
| OID20981 | TNFRSF10B | Tumor necrosis factor receptor superfamily member 10B | 3.330334 | 1.54336 | 7.18635 | 0.00217 | 0.087249 |
| OID20121 | CCL27 | C-C motif chemokine 27 | 2.443983 | 1.379266 | 4.330602 | 0.002201 | 0.087249 |
| OID20339 | CD59 | CD59 glycoprotein | 4.875627 | 1.750568 | 13.57945 | 0.002434 | 0.087249 |
| OID21310 | VNN2 | Vascular non-inflammatory molecule 2 | 2.424991 | 1.36431 | 4.310297 | 0.00254 | 0.087249 |
| OID21316 | CD38 | ADP-ribosyl cyclase/cyclic ADP-ribose hydrolase 1 | 3.301154 | 1.517978 | 7.179037 | 0.002587 | 0.087249 |
| OID21446 | TNFRSF19 | Tumor necrosis factor receptor superfamily member 19 | 2.718183 | 1.415471 | 5.219831 | 0.002667 | 0.087249 |
| OID21531 | MIA | Melanoma-derived growth regulatory protein | 2.652794 | 1.40234 | 5.018264 | 0.002703 | 0.087249 |
| OID20487 | IL1RL2 | Interleukin-1 receptor-like 2 | 2.962859 | 1.45697 | 6.025198 | 0.002706 | 0.087249 |
| OID20321 | RNASET2 | Ribonuclease T2 | 4.395795 | 1.667598 | 11.58733 | 0.002753 | 0.087249 |
| OID21349 | DTX3 | Probable E3 ubiquitin-protein ligase DTX3 | 3.913111 | 1.598105 | 9.581619 | 0.002826 | 0.087249 |
| OID20332 | COL18A1 | Collagen alpha-1(XVIII) chain | 5.517984 | 1.791001 | 17.00063 | 0.002929 | 0.087249 |
| OID20065 | HK2 | Hexokinase-2 | 2.411992 | 1.339117 | 4.344436 | 0.003361 | 0.087994 |
| OID21474 | DCBLD2 | Discoidin, CUB and LCCL domain-containing protein 2 | 3.270152 | 1.476905 | 7.240746 | 0.003483 | 0.087994 |
| OID20964 | TNFRSF6B | Tumor necrosis factor receptor superfamily member 6B | 1.787456 | 1.210308 | 2.639824 | 0.003506 | 0.087994 |
| OID21451 | EDA2R | Tumor necrosis factor receptor superfamily member 27 | 2.330238 | 1.3154 | 4.128026 | 0.003736 | 0.087994 |
| OID20430 | IL13 | Interleukin-13 | 0.50966 | 0.322825 | 0.804626 | 0.003815 | 0.087994 |
| OID20095 | GPR37 | Prosaposin receptor GPR37 | 1.669257 | 1.17723 | 2.366928 | 0.00403 | 0.087994 |
| OID21457 | GFRA1 | GDNF family receptor alpha-1 | 3.061085 | 1.415291 | 6.620716 | 0.004476 | 0.087994 |
| OID20633 | IL16 | Pro-interleukin-16 | 2.214206 | 1.279003 | 3.833226 | 0.004528 | 0.087994 |
| OID21088 | SERPINB1 | Leukocyte elastase inhibitor | 2.160676 | 1.26838 | 3.680698 | 0.004586 | 0.087994 |
| OID20982 | EPHB6 | Ephrin type-B receptor 6 | 3.052178 | 1.409236 | 6.610526 | 0.004654 | 0.087994 |
| OID21142 | RELT | Tumor necrosis factor receptor superfamily member 19L | 2.875001 | 1.383296 | 5.975313 | 0.004665 | 0.087994 |
| OID20154 | LGALS1 | Galectin-1 | 2.878288 | 1.382638 | 5.991834 | 0.004712 | 0.087994 |
| OID21048 | SCARF2 | Scavenger receptor class F member 2 | 3.221448 | 1.43056 | 7.254308 | 0.004735 | 0.087994 |
| OID20460 | WNT9A | Protein Wnt-9a | 3.259544 | 1.433321 | 7.412592 | 0.00482 | 0.087994 |
| OID20344 | DEFA1_DEFA1B | Neutrophil defensin 1 | 2.235643 | 1.277608 | 3.912076 | 0.00483 | 0.087994 |
| OID20943 | SCARB2 | Lysosome membrane protein 2 | 2.711298 | 1.350493 | 5.443297 | 0.005032 | 0.087994 |
| OID20783 | TNFRSF14 | Tumor necrosis factor receptor superfamily member 14 | 3.088915 | 1.40421 | 6.794853 | 0.005047 | 0.087994 |
| OID20286 | CORO1A | Coronin-1A | 1.853613 | 1.20248 | 2.857327 | 0.005188 | 0.087994 |
| OID20399 | CHI3L1 | Chitinase-3-like protein 1 | 1.591916 | 1.148934 | 2.205692 | 0.005199 | 0.087994 |
| OID20525 | EDAR | Tumor necrosis factor receptor superfamily member EDAR | 1.920297 | 1.212243 | 3.041915 | 0.005434 | 0.089938 |
| OID21018 | CD8A | T-cell surface glycoprotein CD8 alpha chain | 2.110297 | 1.243998 | 3.579872 | 0.005611 | 0.090837 |
| OID21059 | LRPAP1 | Alpha-2-macroglobulin receptor-associated protein | 4.550314 | 1.551567 | 13.3448 | 0.005777 | 0.091533 |
| OID20265 | TNFRSF10C | Tumor necrosis factor receptor superfamily member 10C | 2.709892 | 1.329123 | 5.525083 | 0.006092 | 0.094516 |
| OID21043 | FABP5 | Fatty acid-binding protein 5 | 1.837128 | 1.182257 | 2.854743 | 0.006841 | 0.09739 |
| OID21158 | IGFBP4 | Insulin-like growth factor-binding protein 4 | 1.894784 | 1.190895 | 3.014713 | 0.00699 | 0.09739 |
| OID21429 | LRIG1 | Leucine-rich repeats and immunoglobulin-like domains protein 1 | 2.741231 | 1.316587 | 5.707444 | 0.007037 | 0.09739 |
| OID21410 | DPY30 | Protein dpy-30 homolog | 2.24713 | 1.245248 | 4.05509 | 0.007183 | 0.09739 |
| OID21328 | CD302 | CD302 antigen | 2.701614 | 1.308505 | 5.577907 | 0.007211 | 0.09739 |
| OID20998 | BCAN | Brevican core protein | 3.053272 | 1.348994 | 6.910684 | 0.0074 | 0.09739 |
| OID21274 | IGF1R | Insulin-like growth factor 1 receptor | 4.959927 | 1.534212 | 16.03486 | 0.007474 | 0.09739 |
| OID20222 | NECTIN2 | Nectin-2 | 3.146716 | 1.357295 | 7.295261 | 0.007538 | 0.09739 |
| OID20774 | DAG1 | Dystroglycan | 3.028148 | 1.34267 | 6.829438 | 0.007582 | 0.09739 |
| OID21490 | NECTIN4 | Nectin-4 | 2.804499 | 1.309497 | 6.006285 | 0.007956 | 0.09739 |
| OID20737 | LAIR1 | Leukocyte-associated immunoglobulin-like receptor 1 | 2.291786 | 1.24208 | 4.228618 | 0.007962 | 0.09739 |
| OID21023 | SERPINB6 | Serpin B6 | 2.78436 | 1.304428 | 5.943342 | 0.008122 | 0.09739 |
| OID20724 | CD40 | Tumor necrosis factor receptor superfamily member 5 | 2.987943 | 1.326231 | 6.731713 | 0.008259 | 0.09739 |
| OID21064 | JAM2 | Junctional adhesion molecule B | 2.887128 | 1.311825 | 6.354131 | 0.008429 | 0.09739 |
| OID21258 | PLXDC1 | Plexin domain-containing protein 1 | 0.250118 | 0.088932 | 0.703444 | 0.00862 | 0.09739 |
| OID21008 | CDH3 | Cadherin-3 | 2.493314 | 1.259993 | 4.933846 | 0.008698 | 0.09739 |
| OID21461 | SMOC1 | SPARC-related modular calcium-binding protein 1 | 2.876566 | 1.302583 | 6.352479 | 0.008949 | 0.09739 |
| OID20400 | CST3 | Cystatin-C | 2.756923 | 1.288314 | 5.899669 | 0.008984 | 0.09739 |
| OID20071 | VAMP5 | Vesicle-associated membrane protein 5 | 2.514387 | 1.2587 | 5.022758 | 0.009009 | 0.09739 |
| OID21482 | TNFRSF12A | Tumor necrosis factor receptor superfamily member 12A | 2.409706 | 1.242361 | 4.673909 | 0.009267 | 0.09739 |
| OID20410 | C2 | Complement C2 | 3.441781 | 1.355422 | 8.739606 | 0.009332 | 0.09739 |
| OID20856 | BAX | Apoptosis regulator BAX | 1.601154 | 1.121308 | 2.286344 | 0.009599 | 0.09739 |
| OID21101 | GUCA2A | Guanylin | 2.249996 | 1.217833 | 4.156958 | 0.009619 | 0.09739 |
| OID20904 | MAD1L1 | Mitotic spindle assembly checkpoint protein MAD1 | 2.349309 | 1.229644 | 4.488497 | 0.009714 | 0.09739 |
| OID21196 | KIR2DL3 | Killer cell immunoglobulin-like receptor 2DL3 | 1.492677 | 1.101671 | 2.022459 | 0.009743 | 0.09739 |
| OID20704 | CST7 | Cystatin-F | 1.917173 | 1.170326 | 3.140625 | 0.00975 | 0.09739 |
| OID21489 | MDK | Midkine | 2.177616 | 1.206235 | 3.931252 | 0.009819 | 0.09739 |
| OID20213 | SORT1 | Sortilin | 3.343675 | 1.335552 | 8.371192 | 0.009939 | 0.09739 |
| OID20252 | PLA2G1B | Phospholipase A2 | 2.049812 | 1.185442 | 3.544441 | 0.010204 | 0.098086 |
| OID20730 | SMOC2 | SPARC-related modular calcium-binding protein 2 | 2.633476 | 1.257122 | 5.516728 | 0.010273 | 0.098086 |

Estimates (OR(95%CI)) were obtained from logistic regression on individual plasma proteins with lower AVR (≤75^th^ percentile), adjusting for age and sex.

Supplementary Table 10. Proteins mediating the observed association of lower AVR and incident MBI.

|  |  |  | **Path a** | **Path b** | **Direct effect** | **Proportion mediated (%)** |
| --- | --- | --- | --- | --- | --- | --- |
| **OlinkID** | **Assay** | **Description** | **β (SE)** | **β (SE)** | **β (SE)** |  |
| OID21274 | IGF1R | Insulin-like growth factor 1 receptor | **0.12(0.04)** | **1.30(0.62)** | 0.63(0.42) | 19.06 |
| OID21349 | DTX3 | Probable E3 ubiquitin-protein ligase DTX3 | **0.19(0.06)** | **0.83(0.40)** | 0.64(0.42) | 19.22 |
| OID21458 | NCS1 | Neuronal calcium sensor 1 | **0.17(0.05)** | **1.30(0.58)** | 0.57(0.42) | 27.71 |
| OID20095 | GPR37 | Prosaposin receptor GPR37 | **0.38(0.14)** | **0.38(0.18)** | 0.65(0.42) | 17.84 |
| OID20410 | C2 | Complement C2 | **0.10(0.04)** | **1.99(0.87)** | 0.66(0.42) | 24.41 |

Path a: Plasma proteins = AVR + age + sex.

Path b: Incident MBI = AVR + plasma proteins + age + sex.

Exploratory mediation analyses were performed and five plasma proteins showed potential mediation effect on the association between lower AVR (≤75^th^ percentile) and incident MBI. These significant associations did not survive FDR correction. *P<0.05 are highlighted in bold.

**Supplementary Figures**

Supplementary Figure 1. Distribution and jitter dot plots of retinal vascular measures.

Panel A shows the distribution of retinal vascular measures (AVR, CRAE and CRVE). Panels B and C show jittered dot plots by baseline and incident MBI status, respectively, with median and interquartile range reported.

Supplementary Figure 2. Kaplan-Meier curves of incident MBI and AVR quartiles.

AVR quartiles were significantly associated with incident MBI (p-trend = 0.022), with higher risk observed in Q1 and Q2 compared with Q4 (Supplementary Table 5). These quartile-based patterns supported the use of the 75th percentile as a threshold to define lower AVR for subsequent visualization and analyses.
